# Supplementary material for: Single-cell characterization of step-wise acquisition of carboplatin resistance in ovarian cancer
Source: NPJ Syst Biol Appl. 2022 Jun 17;8:20. doi: 10.1038/s41540-022-00230-z (PMC9206019; doi:10.1038/s41540-022-00230-z)
Supplement: Supplementary file 1 — Supplementary Information [file 41540_2022_230_MOESM1_ESM.pdf]

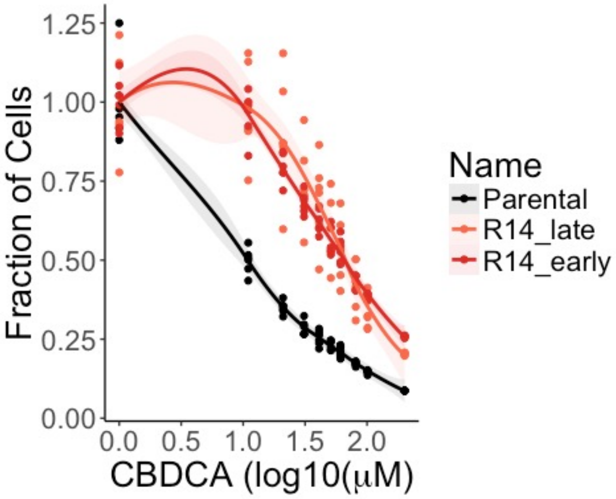

Supplementary Figure 1: Dose Response comparing the drug sensitivity of R14 clones after 36 (R14_early) and 99 (R14_late) doublings after step 15 selection.

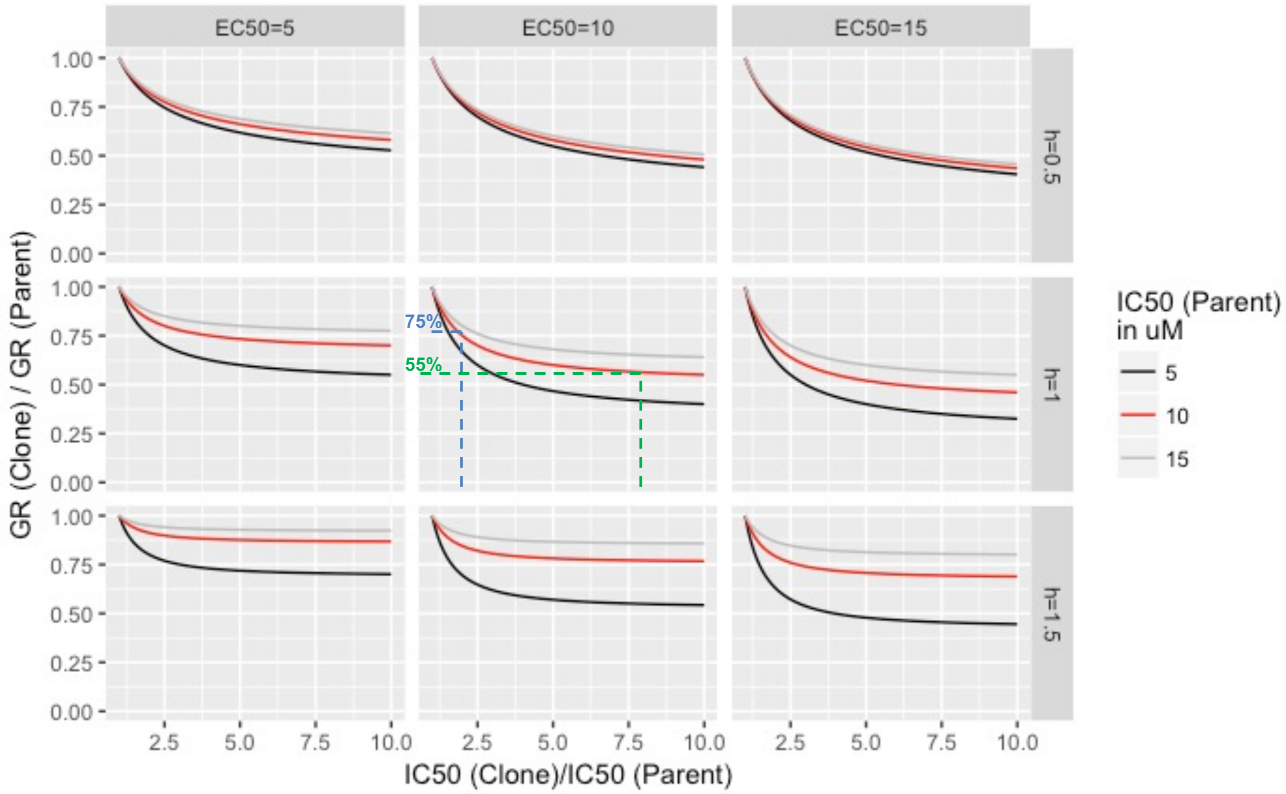

Supplementary Figure 2: Simulated impact of growth rate on resistance. The reduction in growth rate (y axis) is associated with an increase in IC50 (x-axis). The simulations were performed according to Hafner et al (formula IC(c,t) located at http://www.grcalculator.org/grtutorial/). The IC50 of the parental clone (colored lines), hill coefficient (grid rows) and EC50 (grid columns) were tested around the experimental ones observed (IC50=10 μM, h=1, EC50=10 μM, middle panel of the grid, red line). The level of resistance observed in step 5 (blue lines) and step 15 (green lines) is indicated.

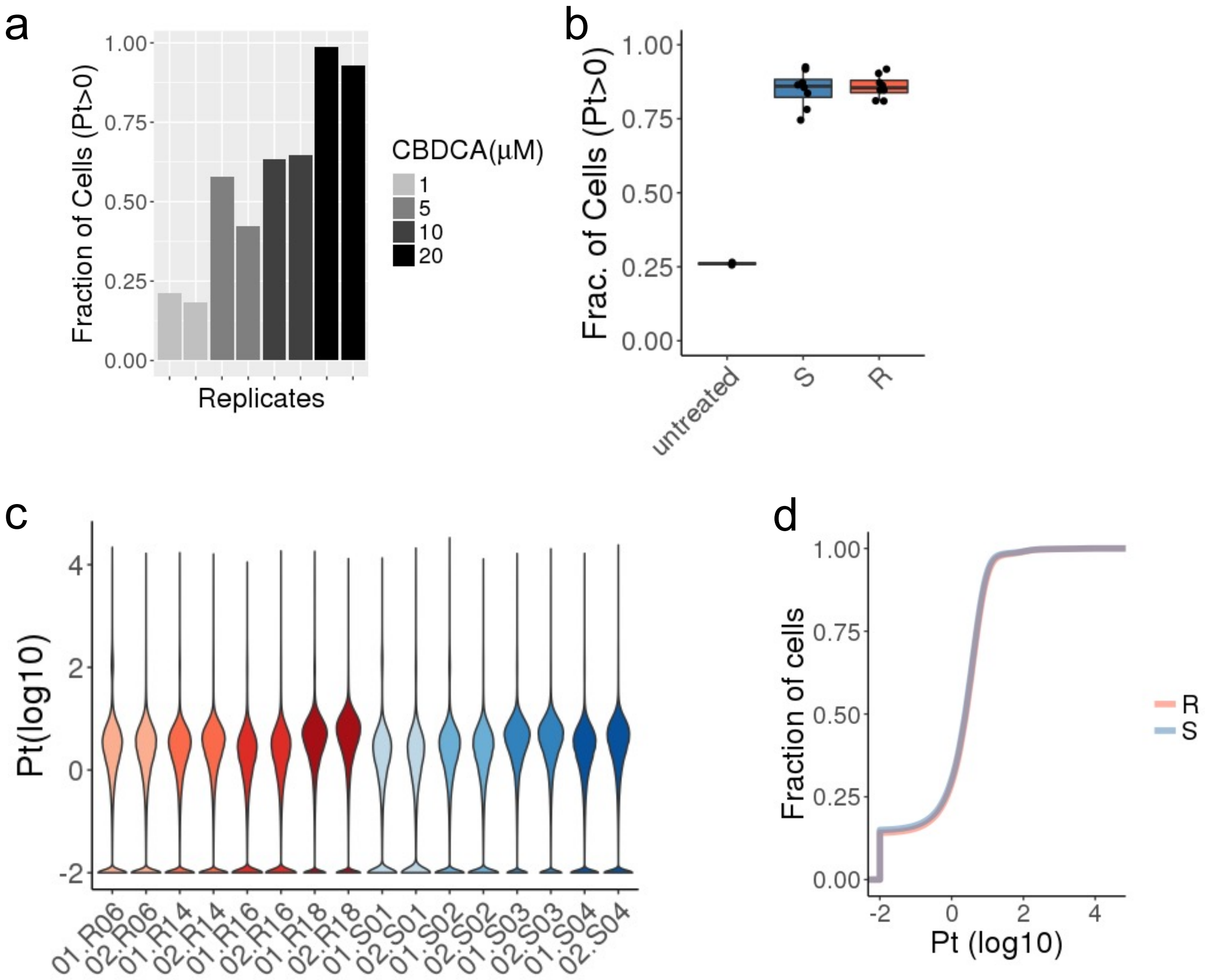

Supplementary Figure 3: Platinum uptake measured by mass cytometry. (a) The fraction of cells form the parental clone with detectable Pt content was measured after 1 h treatment with increasing dose of carboplatin (x axis, grey color gradient). **(b)** Fraction of cells with detectable levels of Pt. Each clone (S and step 15 R clones) and replicate are represented. Boxes represent the top and bottom quartiles of the distribution and whiskers are extended to 1.5 time the interquartile range. **(c)** Violin plot showing the distribution of Pt content across cells from all clones and replicates. **(d)** Cumulative distribution of Pt content between cells from S clones and step 5 R clones and replicates. The Pt negative cells were assigned at 0.01 Pt content for graphical representation (C and D).

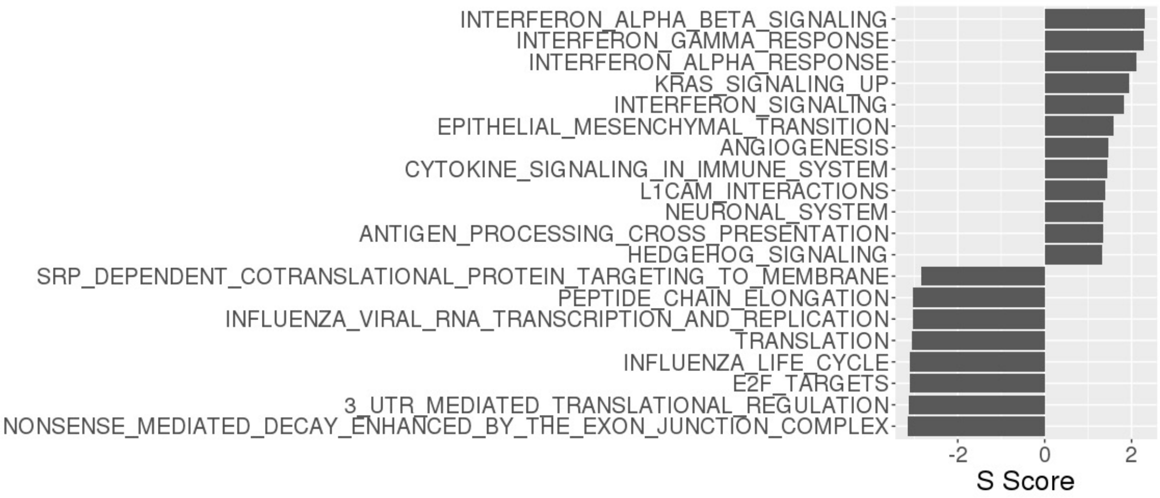

Supplementary Figure 4: Most affected pathways in the resistant clones. Most significantly up or down-regulated processes in CBDCA resistant clones after step 15 selection (q.value<0.05). All Hallmark (N=50) and Reactome (N=674) gene sets from MSigDB were included in the analysis.

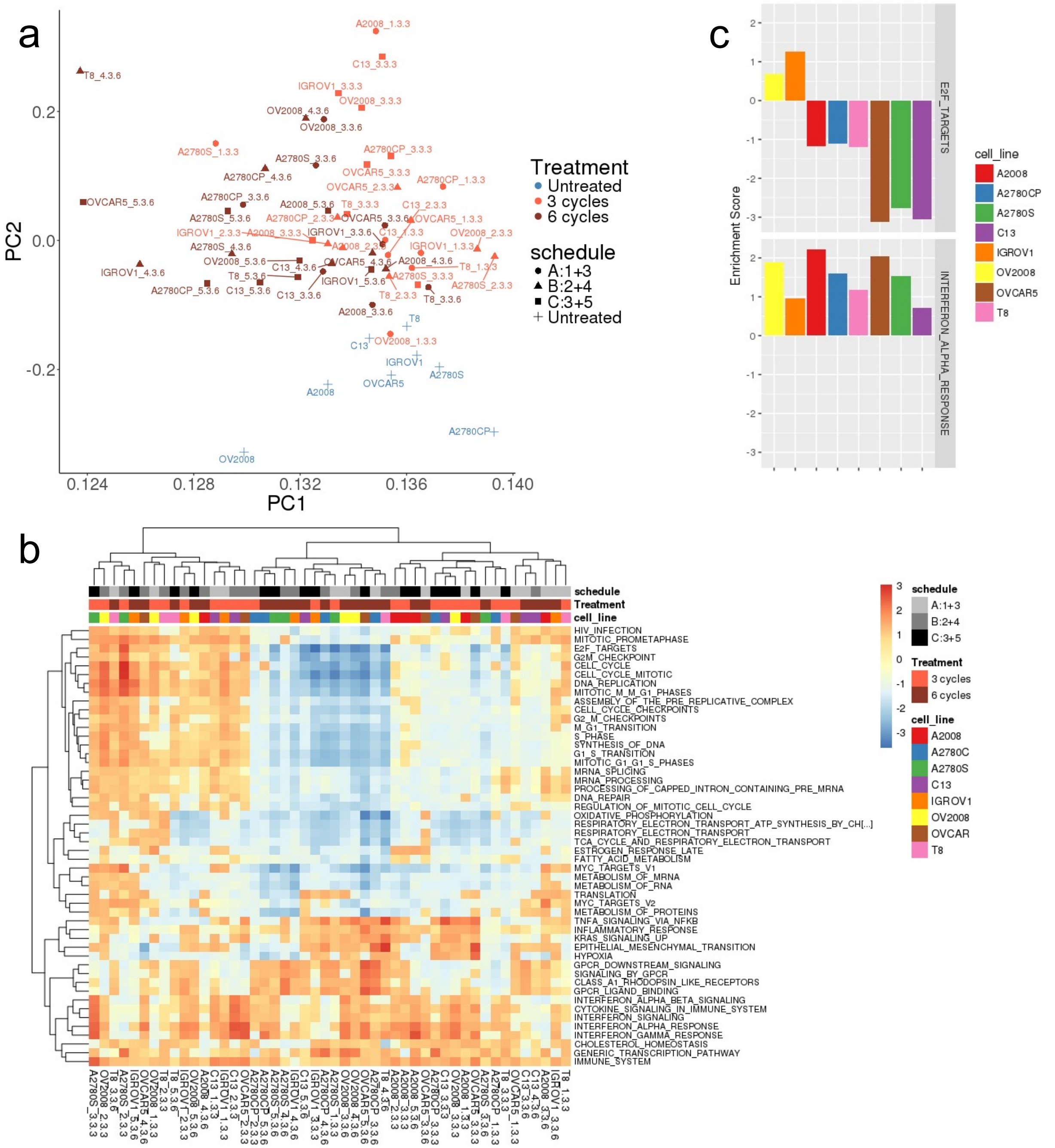

Supplementary Figure 5: Signatures of acquired cisplatin resistance in 8 ovarian cancer cell lines. (a) Principal component analysis of 56 samples from 8 cell lines. Resistant lines were derived from each cell line using 3 treatment schedules of 6 cycles each, increasing cisplatin concentration after 3 cycles. Cells were analyzed after 3 and 6 treatment cycles. Cell line specific variance was corrected for batch effect. See Methods and Marchion et al for details **(b)** Fold change in gene set expression levels compared to all untreated samples. In each treated sample and the set of 8 untreated samples was used to calculate the enrichment score of all Hallmarks and Reactome gene sets from MSigDB. Significant gene sets more than 4 fold induced or repressed in at least one sample are represented. The color scale represents the enrichment score (log scale). **(c)** Enrichment score for two representative gene sets for samples after 6 cycles of treatment schedule C (3 μM then 5 μM)

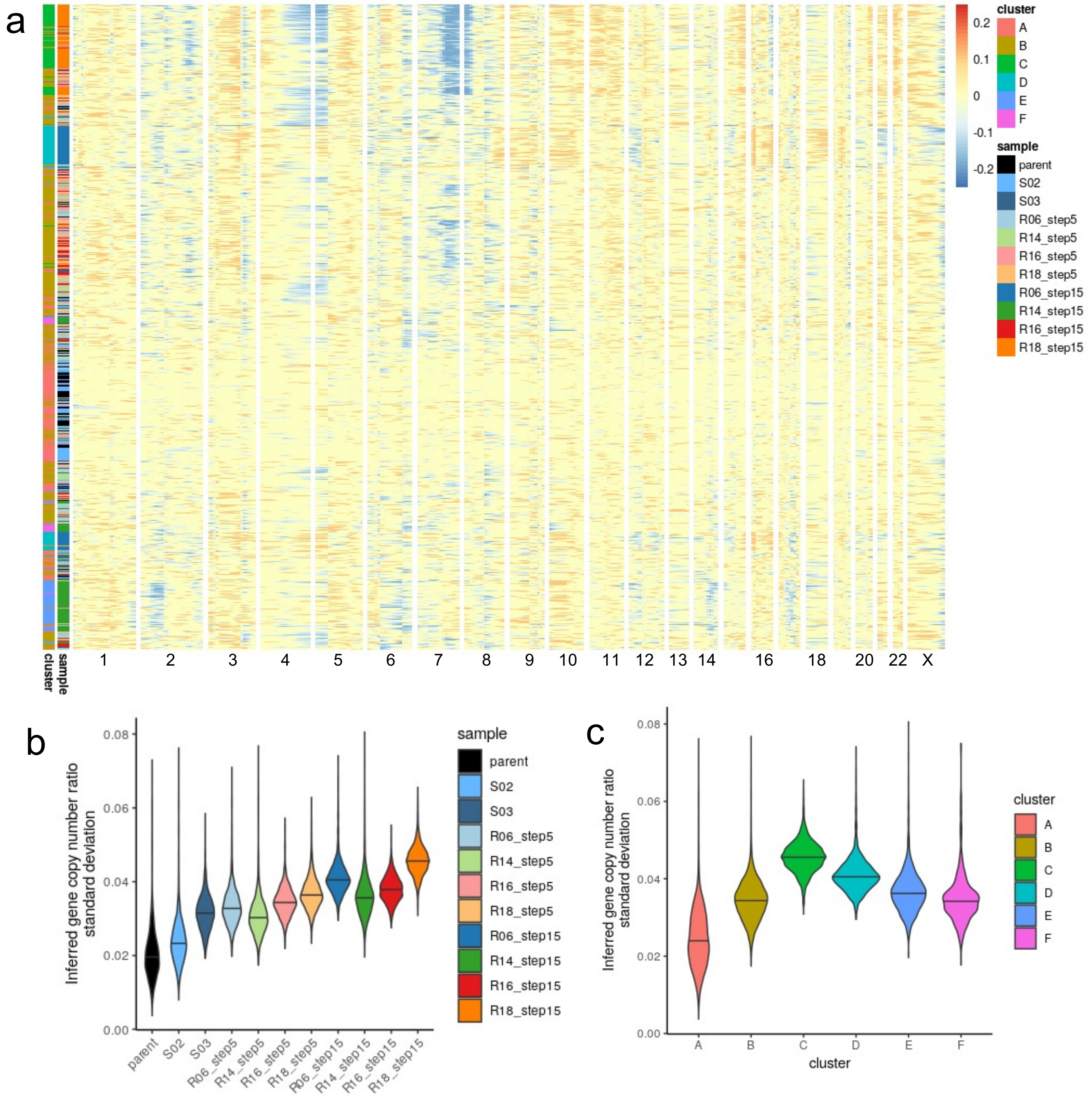

Supplementary Figure 6: Chromosome Copy Number Profile inferred from scRNA-seq expression. **(a)** clustered heatmap of the copy number ratio observed in individual cells (rows) annotated based on their sample and cluster assignment. Genomic interval bins (columns – 10 Mbp windows) are group by chromosome (grouped columns). Values represent the log2 of the average copy number. The COAV3 cells were used as reference group. **(b-c)** Distribution of the standard deviation in gene copy number ratio for all cells in each sample (B) or expression-based cluster (C)

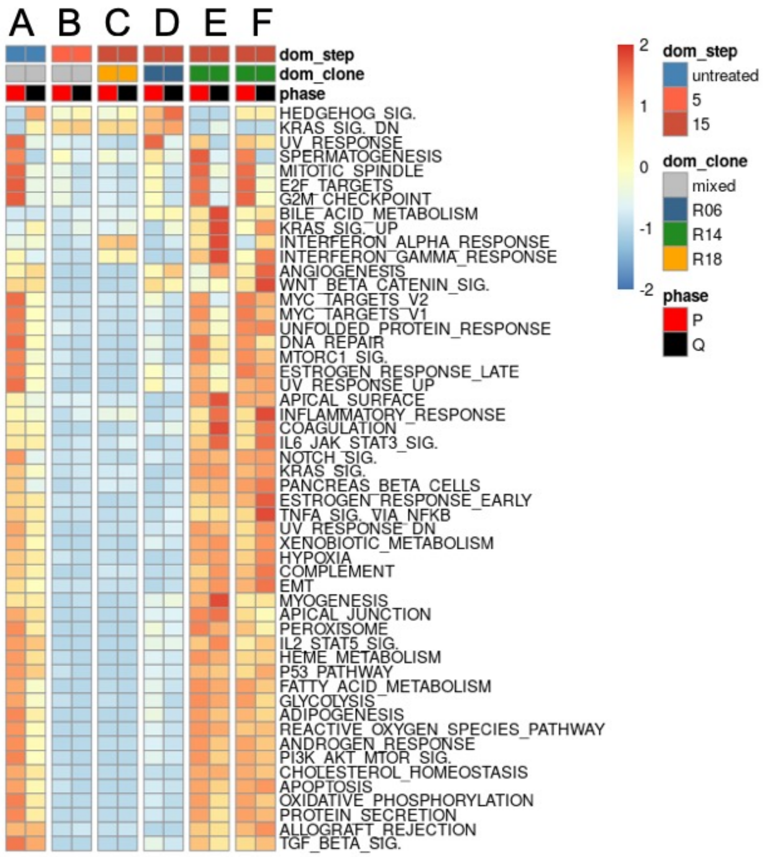

Supplementary Figure 7: Clustered heatmap representing the median gene set enrichment scores (z-score - color-scale) for hallmark gene sets (rows) for all clusters in proliferative (P) or quiescence (Q) phases. Dominant treatment step (dom_step) and clone (dom_clone) in each cluster are indicated.

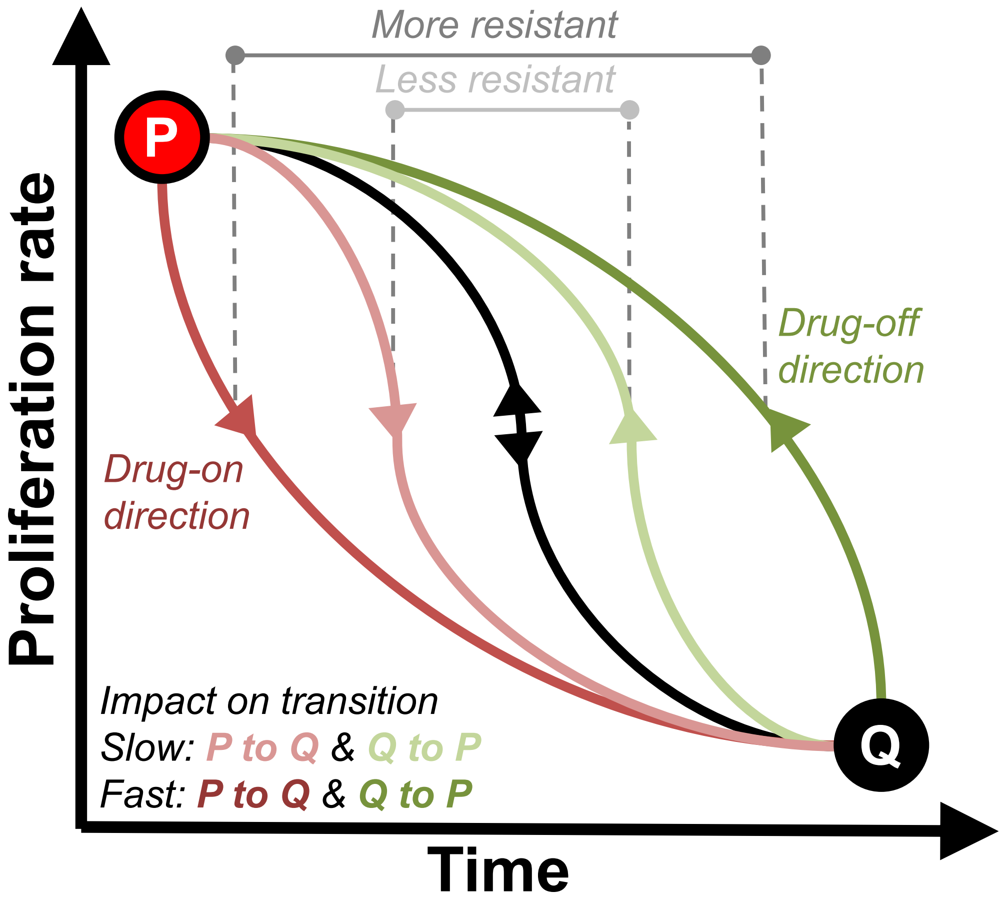

Supplementary Figure 8: A hysteresis model of resistance. Transition between proliferation (P) and quiescence (Q) occurs at each treatment cycle following directional transitions on the hysteresis diagram: drug-on (red, downward paths) and drug-off (green upward paths) transitions. The functional state of the cells in P and Q, different after each cycle, impacts the effectiveness of the transition: light colors and low time-wise slope: slow path, dark color and steep time-wise slope: fast path.

Supplementary Table 1: Copy Number Segments called by CODEX.

Clone	CNV type	chromosome	start	stop	length (kb)	N exons	total	expected	coverage ratio	log	Genes
S01	gain	chr10	225903	1437130	1211	126	9232	7194	0.36	265	ADARB2;DIP2C;GTPBP4;IDI1;IDI2;LARP4B;PRR26;WDR37;ZMYND11
S02	loss	chr20	14066262	16721182	2655	51	3810	4558	-0.26	65	FLRT3;KIF16B;MACROD2;SNRPB2
S02	loss	chr20	16721569	20033488	3312	199	19542	23335	-0.26	327	BANF2;BFSP1;CFAP61;CRNKL1;DSTN;DTD1;DZANK1;KAT14;LINC00493;MGME1;
S02	loss	chr20	20066006	21106921	1041	67	5491	6733	-0.29	122	CFAP61;INSM1;KIZ;RALGAPA2
S02	loss	chr20	21126138	25484748	4359	199	14578	19389	-0.41	654	ABHD12;ACSS1;APMAP;CD93;CST1;CST11;CST2;CST3;CST4;CST5;CST7;CST8;
S02	loss	chr20	25485593	25667185	182	12	892	1099	-0.30	21	NANP;NINL;ZNF337
S01	loss	chr8	48114083	48586432	472	13	1859	2239	-0.27	34	SPIDR
R06	loss	chr5	58120901	58287838	167	9	1193	1440	-0.27	23	PDE4D;RAB3C
S01	gain	chr13	78320681	80911885	2591	52	6192	4542	0.45	269	EDNRB;NDFIP2;POU4F1;RBM26;RNF219;SLAIN1;SPRY2
S01	loss	chr13	101707560	102235705	528	46	2332	3125	-0.42	110	ITGBL1;NALCN
R16	gain	chr2	128335747	131878635	3543	199	36682	31619	0.21	385	AC013269.5;AMER3;AMMECR1L;ARHGEF4;CCDC115;CCDC74B;CFC1;CFC1B;F
S03	loss	chr7	151946959	152143463	197	15	590	872	-0.56	52	KMT2C
R16	gain	chr2	185463620	190044473	4581	199	13613	10963	0.31	297	CALCRL;COL3A1;COL5A2;DIRC1;FAM171B;FSIP2;GULP1;ITGAV;TFPI;ZC3H15;ZN
R16	gain	chr2	190334092	192275992	1942	198	20371	16816	0.28	352	ANKAR;ASNSD1;C2orf88;GLS;HIBCH;INPP1;MFSD6;MSTN;MYO1B;NAB1;NEMP2;
R16	gain	chr2	192280781	192711733	431	10	1572	1288	0.29	29	MYO1B;NABP1;SDPR
S03	loss	chr2	196544782	196681657	137	23	1877	2454	-0.39	74	DNAH7;SLC39A10
S03	loss	chr2	196682401	198267809	1585	199	13365	17234	-0.37	471	ANKRD44;BOLL;C2orf66;CCDC150;COQ10B;DNAH7;GTF3C3;HECW2;HSPD1;HSPE1;HSPE1-MOB4;MARS2;MOB4;PGAP1;PLCL1;RFTN2;SF3B1;STK17B
R06	loss	chr2	197208349	198670120	1462	179	14338	18703	-0.38	555	
S03	loss	chr2	198273032	199011808	739	61	4454	5640	-0.34	135	
R18	loss	chr2	205550863	206058020	507	12	452	652	-0.53	34	PARD3B
R19	loss	chr2	205829848	206058020	228	11	380	572	-0.59	37	

Supplementary Table 2: List novel coding mutations not shared between all clones

Gene	Transcript mutation	Protein mutation	Type	N positive clones		Fisher Pvalue	Description	Canonical Pathway	GO Biological Processes
				Resistant	Sensitive				
AGAP3	c.T94G	p.C32G	nonsynonymous SNV	5	0	0.26	ArfGAP with GTPase domain, ankyrin		GO:0043547 positive regulation of GTPase
CAAP1	c.A307C	p.T103P	nonsynonymous SNV	8	1	0.34	caspase activity and apoptosis		GO:0006915 apoptotic process;GO:0012501
FAM178B	c.C40T	p.P14S	nonsynonymous SNV	0	1	0.38	family with sequence similarity 178		
KAT6A	c.C5738A	p.S1913Y	nonsynonymous SNV	0	1	0.38	lysine acetyltransferase 6A		GO:0043966 histone H3
OAT	c.G418T	p.V140F	nonsynonymous SNV	0	1	0.38	ornithine aminotransferase		GO:0055129 L-proline biosynthetic
PCDH17	c.C2453A	p.P818Q	nonsynonymous SNV	0	1	0.38	protocadherin 17		GO:1904071 presynaptic active zone
SIGLEC5	c.C857A	p.P286H	nonsynonymous SNV	0	1	0.38	sialic acid binding Ig like lectin 5		GO:0043312 neutrophil
SVIL	c.G2355T	p.K785N	nonsynonymous SNV	0	1	0.38	supervillin	(M58)PID AR PATHWAY	GO:0032467 positive regulation of
TLR5	c.C2287A	p.L763I	nonsynonymous SNV	0	1	0.38	toll like receptor 5		GO:0034146 toll-like receptor 5 signaling
C2orf72	c.C364G	p.R122G	nonsynonymous SNV	2	2	0.60	chromosome 2 open reading frame		
LONRF1	c.C958A	p.Q320K	nonsynonymous SNV	7	2	0.66	LON peptidase N-terminal domain		GO:0032436 positive regulation of proteasomal
A2M	c.C1746G	p.H582Q	nonsynonymous SNV	8	3	1.00	alpha-2-macroglobulin	(M183)PID IL6 7 PATHWAY;	GO:0001869 negative regulation of complement
ACAN	c.A1106G	p.D369G	nonsynonymous SNV	1	0	1.00	aggrecan	(M5882)NABA PROTEOGLYCANS;	GO:0042340 keratan sulfate catabolic
ARX	c.C539T	p.A180V	nonsynonymous SNV	8	3	1.00	aristaless related homeobox		GO:0021831 embryonic olfactory bulb
ATXN7L1	c.C969A	p.D323E	nonsynonymous SNV	8	3	1.00	ataxin 7 like 1		
CACNA1E	c.C5806A	p.P1936T	nonsynonymous SNV	8	3	1.00	calcium voltage-gated channel		GO:0050796 regulation of insulin
CBFA2T3	c.C1829T	p.P610L	nonsynonymous SNV	6	3	1.00	CBFA2/RUNX1 translocation partner	(M13)PID ERBB4 PATHWAY	GO:0045820 negative regulation of glycolytic
CCER1	c.A896G	p.E299G	nonsynonymous SNV	1	0	1.00	coiled-coil glutamate rich protein 1		
CILP2	c.G2648T	p.S883I	nonsynonymous SNV	1	0	1.00	cartilage intermediate layer protein 2	(M3008)NABA ECM	
CSMD1	c.C2270A	p.P757Q	nonsynonymous SNV	5	2	1.00	CUB and Sushi multiple domains 1		
DARS	c.A1451G	p.H484R	nonsynonymous SNV	7	4	1.00	aspartyl-tRNA synthetase		GO:0006422 aspartyl-tRNA
EFCAB5	c.C1513T	p.P505S	nonsynonymous SNV	3	2	1.00	EF-hand calcium binding domain 5		
ELAC2	c.A373C	p.I125L	nonsynonymous SNV	1	0	1.00	elaC ribonuclease Z 2		GO:0072684 mitochondrial tRNA 3'-trailer
ERI1	c.C908T	p.A303V	nonsynonymous SNV	8	3	1.00	exoribonuclease 1		GO:0071044 histone mRNA catabolic
FBN1	c.C2501A	p.S834Y	nonsynonymous SNV	1	0	1.00	fibrillin 1	(M212)PID INTEGRIN5 PATHWAY;	GO:2001205 negative regulation of osteoclast
FNDC1	c.A1682G	p.D561G	nonsynonymous SNV	3	1	1.00	fibronectin type III domain containing	(M3008)NABA ECM	
FRG2C	c.129_130del	p.K43fs	frameshift deletion	8	3	1.00	FSHD region gene 2 family member		
FRG2C	c.31_36TAGTGC	NA	nonframeshift substitution	4	2	1.00			
FSTL4	c.G1082A	p.R361K	nonsynonymous SNV	7	4	1.00	follistatin like 4		GO:0048671 negative regulation of collateral
FUT5	c.C582G	p.H194Q	nonsynonymous SNV	8	3	1.00	fucosyltransferase 5		GO:0042355 L-fucose catabolic
GDF7	c.124_125insGGCGGC	p.G42delinsGRR	nonframeshift insertion	1	0	1.00	growth differentiation factor 7	(M5883)NABA SECRETED FACTORS;	GO:2001051 positive regulation of tendon cell
GRIN2C	c.G1463C	p.R488P	nonsynonymous SNV	4	3	1.00	glutamate ionotropic receptor NMDA		GO:0098976 excitatory chemical synaptic
HLA-DRB5	c.169_181GAGAGACACTTCC	NA	nonframeshift substitution	6	3	1.00	major histocompatibility complex,		GO:0031295 T cell costimulation;GO:0050852 T
HSPB9	c.G442C	p.G148R	nonsynonymous SNV	1	0	1.00	heat shock protein family B (small)		
HYAL3	c.T185C	p.L62P	nonsynonymous SNV	2	0	1.00	hyaluronoglucosaminidase 3	(M3468)NABA ECM REGULATORS;	GO:2000355 negative regulation of ovarian
KAT6B	c.G3739C	p.V1247L	nonsynonymous SNV	8	3	1.00	lysine acetyltransferase 6B		GO:0043966 histone H3
KLHL1	c.G1837T	p.D613Y	nonsynonymous SNV	1	0	1.00	kelch like family member 1		GO:0021680 cerebellar Purkinje cell layer
LILRB3	c.1677_1681TTCCC	LILRB3	nonframeshift substitution	2	0	1.00	leukocyte immunoglobulin like		GO:0045671 negative regulation of osteoclast
LOXL4	c.C1602G	p.D534E	nonsynonymous SNV	7	3	1.00	lysyl oxidase like 4	(M3468)NABA ECM REGULATORS;	GO:0006898 receptor-mediated
LRRC19	c.C71A	p.S24Y	nonsynonymous SNV	6	3	1.00	leucine rich repeat containing 19		GO:0046426 negative regulation of JAK-STAT
LRRC75B	c.G41C	p.G14A	nonsynonymous SNV	7	4	1.00	leucine rich repeat containing 75B		
MAGEC1	c.755_757CCC	NA	nonframeshift substitution	5	3	1.00	MAGE family member C1		
MED14	c.G713T	p.G238V	nonsynonymous SNV	1	0	1.00	mediator complex subunit 14		GO:0030521 androgen receptor signaling
MN1	c.C1840G	p.R614G	nonsynonymous SNV	7	4	1.00	MN1 proto-oncogene, transcriptional		GO:0001957 intramembranous
MYH15	c.C5212G	p.Q1738E	nonsynonymous SNV	7	3	1.00	myosin heavy chain 15		GO:0002074 extraocular skeletal muscle
NCAPD2	c.A3527G	p.D1176G	nonsynonymous SNV	6	4	1.00	non-SMC condensin I complex	(M14)PID AURORA B PATHWAY	GO:0010032 meiotic chromosome
NGEF	c.A1G	p.M1V	nonsynonymous SNV	2	0	1.00	neuronal guanine nucleotide	(M83)PID CDC42 REG PATHWAY;	GO:0061002 negative regulation of dendritic
NMUR2	c.C368A	p.T123K	nonsynonymous SNV	1	0	1.00	neuromedin U receptor 2		GO:0043006 activation of phospholipase A2
NOS2	c.T13C	p.W5R	nonsynonymous SNV	4	1	1.00	nitric oxide synthase 2	(M118)PID INTEGRIN A9B1	GO:0031284 positive regulation of guanylate
NTRK3	c.C279G	p.N93K	nonsynonymous SNV	8	3	1.00	neurotrophic receptor tyrosine kinase	(M100)PID SHP2 PATHWAY;	GO:0019056 modulation by virus of host
ODF3	c.C53A	p.P18H	nonsynonymous SNV	8	3	1.00	outer dense fiber of sperm tails 3		GO:0007283 spermatogenesis;GO:0048232
OTUD7A	c.G889T	p.D297Y	nonsynonymous SNV	2	0	1.00	OTU deubiquitinase 7A		GO:0071947 protein deubiquitination involved in
PAM	c.G2220T	p.L740F	nonsynonymous SNV	6	4	1.00	peptidylglycine alpha-amidating		GO:0001519 peptide amidation;GO:0018032
PCDHB8	c.A383G	p.N128S	nonsynonymous SNV	8	3	1.00	protocadherin beta 8		GO:0007156 homophilic cell adhesion via
PHOX2A	c.C763G	p.Q255E	nonsynonymous SNV	7	3	1.00	paired like homeobox 2a		GO:0021623 oculomotor nerve
PIWIL1	c.G2044T	p.A682S	nonsynonymous SNV	2	0	1.00	piwi like RNA-mediated gene		GO:0035093 spermatogenesis, exchange of
PLEKHD1	c.T1216A	p.C406S	nonsynonymous SNV	7	3	1.00	pleckstrin homology and coiled-coil		
PRAG1	c.3496_3497insCGCCCC	p.P1166delinsPPP	nonframeshift insertion	2	1	1.00	PEAK1 related, kinase-activating		GO:0018108 peptidyl-tyrosine
RAET1E	c.T22C	p.S8P	nonsynonymous SNV	7	4	1.00	retinoic acid early transcript 1E		GO:0045954 positive regulation of natural killer
RNF17	c.G595T	p.A199S	nonsynonymous SNV	1	1	1.00	ring finger protein 17		GO:0007286 spermatid
ROBO1	c.T2612A	p.L871Q	nonsynonymous SNV	2	0	1.00	roundabout guidance receptor 1	(M142)PID AJDISS 2PATHWAY	GO:0021836 chemorepulsion involved in
RPTN	c.G2303C	p.R768P	nonsynonymous SNV	2	0	1.00	repetin	(M5883)NABA SECRETED FACTORS;	GO:0070268 cornification;GO:0031424
RSPO2	c.T329C	p.V110A	nonsynonymous SNV	8	3	1.00	R-spondin 2	(M3008)NABA ECM	GO:0042489 negative regulation of
SALL4	c.C380T	p.P127L	nonsynonymous SNV	7	4	1.00	spalt like transcription factor 4	(M223)PID BETA CATENIN NUC	GO:0001833 inner cell mass cell
SLC15A5	c.T97C	p.S33P	nonsynonymous SNV	8	3	1.00	solute carrier family 15 member 5		GO:0015031 protein transport;GO:0015833
SLC37A2	c.A790C	p.I264L	nonsynonymous SNV	8	3	1.00	solute carrier family 37 member 2		GO:0015760 glucose-6-phosphate
SLC6A6	c.C1248A	p.Y416X	stopgain	1	0	1.00	solute carrier family 6 member 6		GO:0015734 taurine transport;GO:0042908
SQSTM1	c.G922C	p.A308P	nonsynonymous SNV	7	3	1.00	sequestosome 1	(M110)PID IL1 PATHWAY; (M128)PID	GO:1900273 positive regulation of long-term
TBC1D22A	c.G190T	p.A64S	nonsynonymous SNV	7	4	1.00	TBC1 domain family member 22A		GO:1902017 regulation of cilium

TH	c.A302T	p.K101M	nonsynonymous SNV	1	0	1.00	tyrosine hydroxylase	(M199)PID P38 MK2 PATHWAY;	GO:0006585 dopamine biosynthetic process
THEMIS2	c.A227G	p.N76S	nonsynonymous SNV	1	0	1.00	thymocyte selection associated family		GO:0050852 T cell receptor signaling
TSPAN16	c.692dupG	p.W231fs	frameshift insertion	6	4	1.00	tetraspanin 16		GO:0007166 cell surface receptor signaling
WDHD1	c.C2359T	p.R787X	stopgain	7	4	1.00	WD repeat and HMG-box DNA		GO:0070829 heterochromatin
ZFC3H1	c.A2531G	p.E844G	nonsynonymous SNV	7	3	1.00	zinc finger C3H1-type containing		GO:0006396 RNA processing;GO:0016070 RNA
ZNF615	c.A767G	p.Q256R	nonsynonymous SNV	1	0	1.00	zinc finger protein 615		GO:0006355 regulation of transcription, DNA-

Supplementary Table 3: Single cell Pt content measured by Mass Cytometry

Clone	Replicate	N cells	N cells with Pt>0	log10(Pt)		
				Mean	SD	Group Mean
R06	1	182799	154826	0.452	0.66	0.434
R06	2	187925	159176	0.375	0.54	
R14	1	187341	161575	0.382	0.56	
R14	2	188674	164508	0.426	0.54	
R16	1	188941	152997	0.285	0.58	
R16	2	187402	151930	0.326	0.56	
R18	1	182658	167601	0.571	0.55	
R18	2	190496	172071	0.626	0.51	
S01	1	187139	146254	0.308	0.61	0.413
S01	2	183219	136607	0.282	0.62	
S02	1	184307	157657	0.377	0.60	
S02	2	186620	156035	0.338	0.55	
S03	1	189050	174895	0.535	0.52	
S03	2	189303	173739	0.540	0.51	
S04	1	189552	165198	0.372	0.54	
S04	2	194450	168067	0.495	0.52	

Supplementary Table 4: Exome sequencing summary statistics

Clone	N or reads	mean coverage depth	Fraction of bp covered 30x or more
S04	61,765,564	77	0.87
S03	55,798,415	68	0.84
S02	62,527,226	76	0.87
S01	55,522,186	68	0.84
R19	53,519,149	65	0.82
R18	58,506,345	68	0.85
R17	58,542,227	71	0.86
R16	55,582,965	68	0.84
R15	61,704,790	76	0.88
R14	65,845,085	81	0.89
R07	62,365,357	77	0.88
R06	66,855,134	80	0.89

Supplementary Table 5: Summary statistics of the single cell RNA sequencing

Sample	N drug free doublings*	scRNAseq batch ID	Estimated Number of Cells	Mean Reads per Cell	Median Genes per Cell	Number of Reads	Valid Barcodes	Reads Mapped Confidently to Transcriptome	Reads Mapped Confidently to Exonic Regions	Reads Mapped Confidently to Intronic Regions	Reads Mapped Confidently to Intergenic Regions	Sequencing Saturation	Q30 Bases in Barcode	Q30 Bases in RNA Read	Q30 Bases in UMI	Fraction Reads in Cells	Total Genes Detected
CAOV3	NA	1	2258	51783	4141	116927569	98.60%	67.20%	72.00%	8.20%	2.50%	17.00%	96.30%	67.30%	96.20%	85.20%	19183
Parent	NA	1	2001	57138	4236	114333459	98.60%	65.50%	70.40%	8.10%	2.50%	17.30%	96.30%	66.70%	96.20%	84.70%	19258
S02	NA	1	2089	44561	3926	93089101	98.50%	63.00%	67.90%	8.40%	2.60%	12.00%	96.20%	63.50%	96.20%	87.40%	19682
S03	NA	1	1495	69191	4786	103441435	98.60%	64.10%	68.90%	8.40%	2.50%	17.30%	96.30%	64.20%	96.20%	86.30%	19282
R06_step15	38	1	2187	43897	4235	96003442	98.50%	67.30%	71.80%	9.10%	2.60%	18.00%	96.30%	66.80%	96.20%	81.20%	19604
R06_step5	24	2	2389	53484	4033	127773590	98.60%	68.60%	73.00%	9.10%	3.00%	28.30%	97.60%	68.90%	97.50%	94.10%	20118
R14_step15	77	1	2836	32814	3540	93063041	98.50%	64.30%	69.40%	7.90%	2.50%	12.60%	96.20%	62.90%	96.20%	84.40%	19657
R14_step5	25	2	2533	52998	3795	134244840	98.60%	66.40%	71.10%	9.60%	3.30%	27.90%	97.60%	68.30%	97.50%	93.50%	20558
R16_step15	67	2	1931	17461	2451	33717516	98.60%	62.30%	66.40%	8.90%	3.00%	8.20%	95.50%	58.40%	95.30%	89.60%	18307
R16_step5	14	2	2364	48123	3670	113765069	98.60%	66.70%	71.40%	9.00%	3.10%	26.50%	97.60%	67.10%	97.50%	90.10%	20204
R18_step15	66	2	2731	10534	1804	28768375	98.60%	61.30%	65.50%	9.30%	2.90%	6.30%	95.50%	55.40%	95.20%	89.60%	17843
R18_step5	14	2	2101	58392	4039	122681684	98.60%	66.40%	71.20%	9.20%	3.10%	28.40%	97.60%	66.80%	97.50%	92.50%	20976

* R clones were still considered stably resistant at the studied stage, supported by Figure S1 (Step 15) or Stordal et al (Step 5).

Reference: Stordal, B., Peters, G. & Davey, R. Similar chromosomal changes in cisplatin and oxaliplatin-resistant sublines of the H69 SCLC cell line are not associated with platinum resistance. Genes. Chromosomes Cancer 45, 1094–1105 (2006)
